# Kangaroos at maximum capacity: health assessment of free-ranging eastern grey kangaroos on a coastal headland

**DOI:** 10.1093/jmammal/gyab022

**Published:** 2021-03-30

**Authors:** Maquel E Brandimarti, Rachael Gray, Fabiola R O Silva, Catherine A Herbert

**Affiliations:** 1 School of Life and Environmental Science, The University of Sydney, JD Stewart Building, Camperdown, New South Wales 2006, Australia; 2 Sydney School of Veterinary Science, The University of Sydney, McMaster Building, Camperdown, New South Wales 2006, Australia

**Keywords:** health, kangaroo, macropod, management, marsupial, overabundant, welfare, wildlife

## Abstract

Sprawling urban development is fragmenting the landscape and native wildlife habitats on the Australian east coast. The impact of this rapid urbanization on wildlife health is largely unknown. This study surveyed the health of a high-density (5.4 individuals per ha) population of eastern grey kangaroos (*Macropus giganteus*) affected by urban encroachment and prolonged drought. Blood parameters (hematological and serum protein), trace element and heavy metal concentrations, and parasite counts (fecal worm egg counts, ticks, and mites) are reported for a sample of ≤ 54 kangaroos at Look at Me Now Headland, New South Wales, Australia. These parameters were compared to lower density kangaroo populations from other sites in New South Wales. We found the health and welfare of this population to be severely compromised, with nonregenerative anemia and nutritional deficiencies evident. Our results indicate that high-density kangaroo populations isolated by urban encroachment are at significant health risk. To prevent further decline in this population’s health, we discuss management strategies that could be employed, concurrent with ongoing health and disease monitoring, to mitigate the poor health outcomes in this population. We conclude that it is essential to retain habitat connectivity when altering land use in areas with resident kangaroo populations if managers are to maintain healthy populations.

Humans have changed the natural environment and altered the dynamics of wildlife populations (Foster et al. 2002). This can lead to an increase in density of some species (Ditchkoff et al. 2006), up to the point at which they are considered “overabundant.” These populations reduce natural diversity, affect human life or livelihood, and affect the fitness of individuals within the overabundant population itself (Adderton Herbert 2004). While most studies have focused on the negative impacts of overabundant species on the ecosystem they inhabit, less is known about the impacts on the overabundant species themselves.

Overabundant populations often are visually perceived as flourishing, and the immediate impacts that increasing density can have on animal health, welfare, and long-term population viability often are overlooked (Garrott et al. 1993). High-density populations are exposed to an array of physiological stressors that can affect an individual’s diet, reproductive success, disease status, welfare, and survival (Scott 1988; Gortázar et al. 2006). Animals at high density can overexploit food resources (McCarthy 1996) causing malnutrition, starvation, and mortality. This can increase their susceptibility to disease and parasitism, resulting from greater intraspecies contact and opportunities for disease transmission (Gortázar et al. 2006). For urbanized native species, these stressors are exacerbated by habitat fragmentation, infrastructure expansion, and motor vehicle collisions (Brunton et al. 2019). As the urban environment expands and continues to distort and disrupt native populations and their habitats, there is a growing need to explicate the indirect costs of these changes to wildlife health, welfare, and population resilience.

Wildlife health surveillance is increasingly important due to ongoing biodiversity loss and increasing threats of zoonotic disease (Ryser-Degiorgis 2013; Han et al. 2016). Wildlife health investigations may require species-specific baseline health parameters for long-term health monitoring of populations. If species-specific baseline hematology and biochemical reference intervals (RIs) are known, the potential to gather physiological data and identify disease can be improved (Maceda-Veiga et al. 2015). The number of individuals in a population that fall outside of the RI provides a quantifiable measure of the impact of parasites, disease, and physiological stressors such as malnutrition (Huber et al. 2017). Blood samples also can enable measurement of stress-related parameters, which are intimately linked to animal health and welfare (Huber et al. 2017). Classical measures of stress include glucocorticoid concentration (Sheriff et al. 2011); however, there is a mounting body of evidence advocating the superiority of using the immune system as an indicator of stress (Davis et al. 2008; Huber et al. 2017). Specifically, the neutrophil to lymphocyte ratio (N:L) can be used as a proxy measure for stress as a result of characteristic changes in the white blood cell (WBC) profile within 4–8 h of exposure to a stressor (Davis et al. 2008; Schultze 2010; Huber et al. 2017). The occurrence of declining health status and/or increased incidence of disease and biomarkers of stress are sensitive indicators of environmental and ecological change for a species (Scott 1988) and can be used to direct management efforts. This is particularly important for overabundant native species in which management often involves controversial techniques such as culling (Descovich et al. 2016).

Eastern grey kangaroos (*Macropus giganteus*, hereafter kangaroos) are a common macropod species with a wide geographic distribution along eastern and southeastern Australia (Coulson 2008). Overabundant kangaroo populations commonly occur on the urban fringes (peri-urban areas) because large-scale movement of animals is uncommon and largely constrained by infrastructure development (Coulson et al. 2014). Kangaroos have been able to persist in these often-isolated pockets of habitat because they actively use urban green spaces, remnant forests, and land cleared for livestock grazing (Coulson et al. 2014). Despite the apparent success of kangaroos, peri-urban landscapes are becoming increasingly fragmented and populations isolated (Brunton et al. 2019). Kangaroo “die-off” events have been described in overabundant kangaroo populations in the Australian Capital Territory (Portas and Snape 2018) with subadult animals primarily affected and at greater risk of starvation when compared to adults. Blood sampling revealed anemia and hypoalbuminemia in a number of animals, while necropsy investigations reported minimal fat reserves in affected individuals (Portas and Snape 2018). Kangaroo populations at unsustainably high densities also are at risk of disease outbreaks, such as oral necrobacillosis (“lumpy jaw”), which can cause severe emaciation and death by starvation (Borland et al. 2012). However, apart from isolated “die-off” events and accounts of disease in enclosed populations, the potential health impacts on peri-urban-dwelling kangaroos largely are unknown (Brunton et al. 2019).

This study describes the health status and density of a population of peri-urban kangaroos from a coastal headland in New South Wales facing ongoing habitat fragmentation, human encroachment, and affected by prolonged drought (NSW DPI 2020). This paper reports and compares the results of hematological and serum protein analyses, parasite counts (gastrointestinal worms, ticks, and mites), and trace element and heavy metal concentrations from kangaroos at Look at Me Now Headland (LAMN) and lower density kangaroo populations elsewhere. Blood parameters of kangaroos sampled at LAMN are compared to established RIs for the species (Brandimarti et al. 2020). In addition, the influence of biotic (sex and maturity) and abiotic (season, site, and rainfall) factors is examined.

## Materials and Methods

### Primary study site (LAMN)

**Fig. 1.**
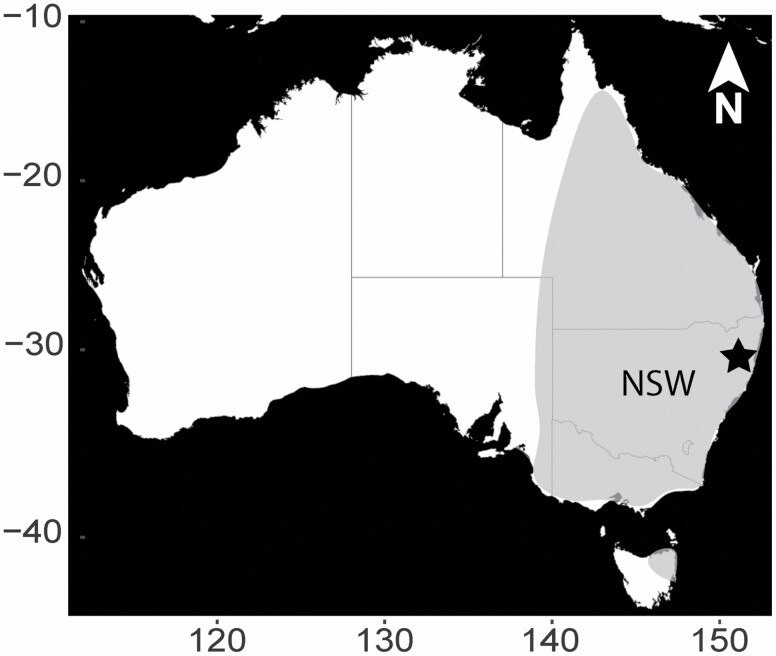
Location of the eastern grey kangaroo (*Macropus giganteus*) population sampled from 2017 to 2019 at Look at Me Now Headland (LAMN, star) in New South Wales, Australia. Degrees of latitude on the y-axis and longitude on the x-axis. Gray shading represents the approximate distribution of eastern grey kangaroos in Australia. Map sourced from Stamen Design, under CC BY 3.0. Data by OpenStreetMap, under ODbL.

The study site, LAMN (30.177°S, 153.189°E) is a headland bordering the coastal town of Emerald Beach, 15 km N of Coffs Harbour, New South Wales (Fig. 1). The headland is part of the Moonee Beach Nature Reserve and is a key area for the conservation of endangered *Themeda*-grassland and threatened flora species such as *Zieria prostrata*; but it also is home to a number of free-ranging macropods, including kangaroos and red-necked wallabies (*Notamacropus rufogriseus*—Hunter and Hunter 2019; Henderson et al. 2018). Previous bimonthly direct counts of kangaroos undertaken in 2016 identified LAMN as a “kangaroo hotspot” with between 2.25 and 4.87 individuals per ha (Henderson et al. 2018).

### Population counts at LAMN

The total population counts undertaken in this study in February 2018 and February 2019 were a continuation of a previous study (Henderson et al. 2018). A similar methodological approach was adopted to permit comparison of counts over time. Counts were undertaken in the late afternoon or early morning, a time of increased kangaroo activity. A total count approach (Inwood et al. 2008) was adopted in which at least two people simultaneously surveyed on foot from walking tracks on both LAMN and the adjacent Damerells Headland, and the adjoining walking tracks, within a 30-min time frame (Supplementary Data SD1). All kangaroos and red-necked wallabies were counted, including young-at-foot (YAF) and excluding dependent pouch young (solely in the pouch). Three counts were carried out on consecutive days in 2018 and 2019 to determine the precision of the count. Total counts were divided by the size of the survey area in hectares (ha), measured using Google Earth to give an average animal density over the study time frame. Precision estimates are presented alongside the means as the coefficient of variation (*CV*).

### Sample collection and parasitological analyses

Adult and subadult kangaroos (*n* = 21 males and *n* = 33 females) were immobilized at LAMN between 2017 and 2019 using Zoletil 100 (Virbac Pty Ltd, Milperra, New South Wales, Australia) with a fixed dose of 65 mg for YAF, 125 mg for females or subadult males and females, and 250 mg for adult males. A 1 ml (or 0.5 ml for YAF) intramuscular injection of Zoletil was administered using a pole syringe or CO_2_-powered projector (X-Calibre, Pneudart Inc., Williamsport, Pennsylvania). Kangaroos were followed from the time of drug administration to the time of immobilization, then transferred into a soft cloth capture bag for transportation to an onsite processing area within 1 km of the capture location. Sex, pes and leg length (Poole et al. 1982), body mass (to nearest 0.01 kg, Wedderburn Digital Hanging Scales, Model: WS603), and female reproductive status (teats not everted [i.e., subadult], pouch young present, lactating but no young present in the pouch [i.e., dependent YAF], or teats everted but no pouch young [i.e., nonreproductive but sexually mature]) were recorded during processing. Male maturity was determined by leg length; male kangaroos with a leg length > 52.3 cm were deemed sexually mature (Poole 1973; Poole et al. 1982). While immobilized, each kangaroo was given a unique combination of colored tags in their left/right ear (“mini” and “button” sheep tags applied using the identiplier applicator [Allflex Australia Pty Ltd, Capalaba, Queensland, Australia]) to allow identification of each animal. Kangaroos were examined clinically for visible injuries, and body condition was scored as “poor,” “thin,” “good,” or “excellent,” based on palpation of the fat cover and muscle mass of the vertebral spinous processes and hip bones (Johnston et al. 1997; Edwards et al. 2013). Muscle mass on the rump also was visually assessed and recorded for animals that were in “thin” or “poor” condition. All body condition assessments were carried out by the same observer (RG). A subjective scoring system was used to assess condition at LAMN because it is nondestructive and can be used to compare animals across populations for future investigations. Tick counts (*n* = 287 individuals, including lower density populations for comparison) were made at six locations on the body (inguinal, axilla, bilateral eyes and ears). An 8 cm × 8 cm square quadrant was placed under either the left or right axilla and left or right inguinal region and all ticks within the quadrant were counted. Bilateral counts of ticks on the perimeter of the eye and on the inner and outer surface of the pinna also were carried out. Total tick count per kangaroo was obtained by summing the number of ticks from all six sites. The presence or absence of mites was recorded following examination of the ears, eyes, axilla, inguinal, and pouch (females) regions (*n* = 325, including lower density populations for comparison). Additional anecdotal observations were reported, including any associated skin lesions, typically noted as crusting of the skin, swelling, redness, or discharge. Heart rate, respiratory rate, capillary refill time, and the color of oral and urogenital mucous membranes were recorded throughout the period of sedation. Up to 6 ml of whole blood was collected from the lateral caudal vein, approximately 30 min after administration of Zoletil 100, using a 23-gauge butterfly catheter (Surflo winged infusion set, Terumo Australia Pty Limited, Macquarie Park, New South Wales, Australia) attached to a 5-ml syringe (Luer slip, Terumo Australia Pty Limited, Macquarie Park, New South Wales, Australia). Blood was immediately transferred into a 5-ml serum tube (BD Vacutainer SST II Advance tube, Becton Dickinson and Company, North Ryde, New South Wales, Australia), 1.3-ml EDTA tube (Becton Dickinson and Company, North Ryde, New South Wales, Australia), and trace element tube (BD Vacutainer K2 EDTA, Becton Dickinson and Company, North Ryde, New South Wales, Australia). One to two drops of fresh blood were applied to a glucose strip (Freestyle optimum glucose strips, Abbott, Alameda, California) and blood glucose determined using a handheld glucose-monitoring device (Freestyle Optium Neo Blood Glucose Monitoring System, Abbott, Alameda, California). Whole blood was gently inverted and stored on an ice brick in an insulated box prior to processing. Whole blood in serum tubes was centrifuged for 10 min at 3,000 rpm (LW Scientific E8C-08AF-150P Porta-Fuge Portable 12 Volt Centrifuge). Serum was stored at −20°C after separation (LW Scientific, Lawrenceville, Georgia). Two blood smears were made within 1 h of blood collection using whole blood preserved in EDTA. Blood smears were air-dried, fixed in methanol, and stained with Diff Quik (Lab Aids Pty Ltd, North Narrabeen, New South Wales, Australia) for differential WBC counts or brilliant cresyl blue (Sigma-Aldrich, Castle Hill, New South Wales, Australia) for reticulocyte counts. Two hundred microliters of whole blood preserved in EDTA was mixed with 200 μl of Streck Cell Preservative (Streck, Omaha, Nebraska) and stored at 4°C prior to automated hematological analysis. After processing, kangaroos were placed in a quiet location within observation distance from the processing site and monitored remotely for up to 2.5 h from time of immobilization. If the animals were mobile before 2.5 h, they were followed at a distance for ongoing observation. During processing or recovery time a fresh fecal sample was usually obtained from each individual from the substrate around the recovery site. If an animal did not defecate during recovery, a fecal sample could be collected opportunistically while the animal was grazing. Fecal samples were stored on an ice brick in an insulated box or in a refrigerator prior to processing (within 48 h). Gastrointestinal worm burdens were estimated (*n* = 418, including lower density populations for comparison) using quantitative estimates of fecal worm eggs per gram of feces (EPG). To determine EPG, a modified McMaster technique was employed whereby 3 g of feces were placed in 60 ml of saturated salt solution (Gibbons et al. 2005). After homogenization a sieve was inserted, and a sample was loaded into two chambers of a McMaster slide. All worm eggs were counted, and the number of eggs multiplied by 40 (1:20 dilution) to obtain an EPG.

All animal captures followed the guidelines of the American Society of Mammalogists (Sikes et al. 2016) and received animal ethics approval from The University of Sydney, 2016/1062 and 2015/917. Animal collection was performed under relevant permits from all New South Wales, Victorian, and Australian Capital Territory governments (scientific license number: SL100961 and SL102148).

### Automated hematological and protein analyses

EDTA whole-blood samples preserved in Streck were analyzed using a Sysmex XN1000i automated hematology analyzer (Roche diagnostics, North Ryde, New South Wales, Australia) at the Veterinary Pathology Diagnostic Services (VPDS), The University of Sydney, Camperdown, New South Wales, Australia, within 7 days of blood collection (after Brandimarti et al. 2020). The following parameters were determined: hematocrit (HCT; l/l), hemoglobin (HGB; g/l), total red blood cell count (RBC; ×10^12^/l), total WBC count (×10^9^/l), platelet count (PLT; ×10^9^/l), and nucleated RBC count (NRBC; ×10^9^/l). Values were doubled to account for dilution with cell preservative and any hemolyzed samples were excluded from the study. Mean cell volume (MCV; fl; [HCT/1,000]/RBC), mean cell hemoglobin (MCH; pg; [HGB/RBC]), and mean corpuscular hemoglobin concentration (MCHC; g/l; [HGB/HCT]) were calculated manually. One hundred WBCs were differentiated on stained blood smears to determine the percentage of neutrophils, lymphocytes, eosinophils, monocytes, and basophils. Absolute counts for each WBC were determined by multiplying the percentage of each WBC type (% from the smear) by the total WBC count determined by the automated analyzer (×10^9^/l). The relative proportion of N:L was determined by dividing absolute neutrophil counts by absolute lymphocyte counts. Reticulocyte counts (RET; ×10^9^/l) were carried out by estimating the number of reticulocytes per 1,000 RBCs on prepared blood smears.

Frozen serum was thawed at room temperature before analyses. Albumin (g/l), total serum protein (TSP; g/l), and globulin (g/l) concentrations were determined using the Konelab Prime 30i analyzer (Thermo Fisher Scientific, North Ryde, New South Wales, Australia) at VPDS, within 7 days of blood collection.

### Trace element and heavy metal analyses

Blood trace element and heavy metal concentrations were determined in whole blood (*n* = 41, including lower density populations for comparison) using the Ultra-Mass Spectrometer System (ICP-MS, Varian Australia Pty Ltd, Mulgrave, Victoria, Australia) at a commercial laboratory (Department of Chemical Pathology, Royal Prince Alfred Hospital, Camperdown, Sydney, New South Wales, Australia). Blanks were regularly analyzed during the sample run to ensure no contamination. Precision and accuracy of analyses were determined by comparing results against certified values (if available), determined using standard material Normal Range Trace Element Serum Toxicology Control (UTAK Lot # 66816, UTAK Labs Inc., Valencia, California) as per laboratory standard operating procedures.

### Comparison study sites

An additional larger data set was used to compare health parameters from a range of sites. This larger data set contains observations from up to five comparative kangaroo populations, described briefly below and previously by Brandimarti et al. (2020). Nelson Bay Golf Course (NBGC; 32.728°S, 152.150°E) is a peri-urban free-ranging population in Port Stephens, New South Wales, Australia, with a population density ranging from 1.88 (2015) to 1.21 (2018) individuals per ha. Vegetation is dominated by pastoral grass species and dry sclerophyll forest. Samples were collected from NBGC between 2015 and 2019. Darlington Park (DP; 30.048°S, 153.191°E) is a free-ranging coastal population in Arrawarra, New South Wales, Australia, with a density of 1.44 individuals per ha. The site is a mixture of caravan park, golf course, private farmland, and coastal bushland. Samples were collected at DP between 2017 and 2019. Heritage Park (HP; 30.183°S, 153.149°E) is a semirural free-ranging population inhabiting a new housing estate and private farmland in Moonee Beach, New South Wales, Australia, with a density of 1.23–1.52 individuals per ha (Henderson et al. 2018). The vegetation at HP consists of grazing pasture, ornamental grasses, and wet and dry sclerophyll forests. Samples from HP were collected in 2017. Ainslie Majura Kangaroo Management Unit (AM; 35.274°S, 149.165°E) is a semirural free-ranging population located within Mount Ainslie Nature Reserve, Canberra, Australian Capital Territory, Australia. The kangaroo density in the broader management area was 1.69 individuals per ha. This site is subject to ongoing population management via routine culling, due to the impacts of high density kangaroos on natural values. The vegetation at AM is dominated by natural temperate grassland and box-gum grassy woodland. Sampling was carried out in 2018. Woolgoolga (WGA; 30.102°S, 153.185°E) is a semirural free-ranging population bordering the town of Woolgoolga, New South Wales, Australia. Vegetation at WGA consists of pastoral grass species and dry sclerophyll forest. The population density at this site is unknown; however, the density of a nearby population (Safety beach golf course) is 1.57–2.32 individuals per ha (Henderson et al. 2018). Samples were collected from WGA in 2019.

### Statistical Analyses

#### Comparison of blood analytes to species-specific RIs and rainfall

Blood analytes were partitioned based on age (adult and subadult) as recommended for interpretation of species-specific RIs (Brandimarti et al. 2020). For both subgroups, each analyte from an individual was compared to the upper and lower limit of the relevant RI (Brandimarti et al. 2020). Each value was classified as residing within the RI range or residing above or below the upper and lower limit. The number of animals in each classification was converted to a percentage of the total sample group at LAMN. Analytes with < 2.5% (nonparametric 95% confidence intervals, *CI*s) or < 5% (robust 90% *CI*s) of the population falling outside the central RI could indicate random error and are not uncommon, even for healthy populations (Friedrichs et al. 2012; Brandimarti et al. 2020). Blood analytes were described using median values with 95% *CI*s to allow for comparison between subgroups and because most analytes were not normally distributed (Shapiro–Wilk normality test). All further analyses were carried out using R version 3.5.3 statistical software (R Core Team 2017). To determine the effect of rainfall on blood parameters, linear mixed-effect models (lmm) using the “lmer” function in the “lme4” package were used (with year and animal ID as random factors), and rainfall as a fixed factor. Rainfall was selected due to its role in driving variation of blood parameters (Brandimarti et al. 2020). Accumulated rainfall (mm) data from the month of sample collection were obtained from the nearest weather station (BOM 2020). Where parameters were normally distributed, a one-way analysis of variance (ANOVA) function in the “car” package then was used to test (Type II Wald chi-squared) analysis of deviance on models to determine *P*-values. For parameters that were not normally distributed, either a square, log, or cube root transformation was undertaken before analysis using ANOVA. If normality could not be established, a Kruskal–Wallis test was applied.

#### Reticulocyte count and N:L ratio analyses

Raw reticulocyte counts are reported because counts were too low for descriptive statistics. To determine the effects of biotic and abiotic factors on N:L ratios (*n* = 286, including lower density populations for comparison) lmm was used. Prior to analysis, a log transformation was carried out and normality and collinearity assessed using the Shapiro–Wilk normality test and variance inflation factor (< 2), respectively. The initial factors for all models were selected from relevance in the literature (Turner et al. 2012; Fancourt and Nicol 2019) and previous work (Brandimarti et al. 2020). Factors for N:L ratio model selection are presented in Table 1. A stepwise method was used to select fixed factors for the model, with the best fit used for final analyses. Factors were added individually, and the model fit was assessed using the Akaike Information Criterion. Residual plots were evaluated to ensure random distribution of error. The ANOVA function then was used to test (Type II Wald chi-squared) analysis of deviance on models to determine *P*-values. For significant factors with more than one level, estimated marginal means were compared to determine significance among levels using the “emmeans” package.

**Table 1.** Model factors for parasite abundance (fecal worm egg counts and ticks), presence–absence (mites), and relative proportion of neutrophils to lymphocytes (N:L ratio) in eastern grey kangaroos (*Macropus giganteus*) from Look at Me Now Headland (LAMN), Nelson Bay Golf Course (NBGC), Darlington Park (DP), Heritage Park (HP), and Ainslie Majura Kangaroo Management Unit (AM). A stepwise method was used to select fixed factors.

Response variable	Abiotic fixed effects	Biotic fixed effects	Random effects
Log_N:L	Site and rainfall	Maturity and sex	ID^b^, year
EPG^a^	Site and rainfall	Maturity and sex	ID^b^, year
Total tick burden	Site	Sex	ID^b^, year
Mite prevalence	Site, rainfall, and season	Sex	ID^b^, year

^a^Eggs per gram of feces, ^b^animal identification.

#### Trace element and heavy metal analyses

Trace and heavy metal analytes were described using median values with 95% *CI*s because most analytes were not normally distributed (Shapiro–Wilk normality test). Values from the sample group at LAMN were qualitatively compared to two separate populations of low density kangaroos (WGA and NBGC). Statistical comparisons were not undertaken due to the small sample size at these additional sites (WGA = 2 and NBGC = 5).

#### Parasitological analyses

To ensure observations were random before proceeding to analyses, separate time series plots of EPG and total tick counts were visually examined and determined to be random. Initial exploration of parasite data revealed that the variance was considerably higher than the mean, precluding the use of a Poisson distribution on this data set (Ver Hoef and Boveng 2007). To determine the biotic and abiotic factors driving parasite abundance, analyses of EPG and total tick counts were carried out using generalized linear mixed-effect regression models (glmm) using the “glmer.nb” function in the “lme4” package. To determine mite presence–absence, a glmm was used with a binomial distribution and log link function. Prior to all analyses, collinearity was assessed using variance inflation factor and found to be < 2. The stepwise method of model selection, ANOVAs, and estimated marginal means (where appropriate) were repeated as above to generate a *P*-value. Selected factors for EPG, tick, and mite analyses are presented in Table 1.

## Results

### Population Count at LAMN

The mean kangaroo density at LAMN in February 2018 and February 2019 was 5.4 (*CV* = 11.3%) individuals per ha. The mean red-necked wallaby density was 0.8 (*CV* = 57.3%) individuals per ha.

### Hematological, Glucose, and Serum Protein Analyses

#### Comparison of blood analytes to species-specific RIs and rainfall

**Fig. 2.**
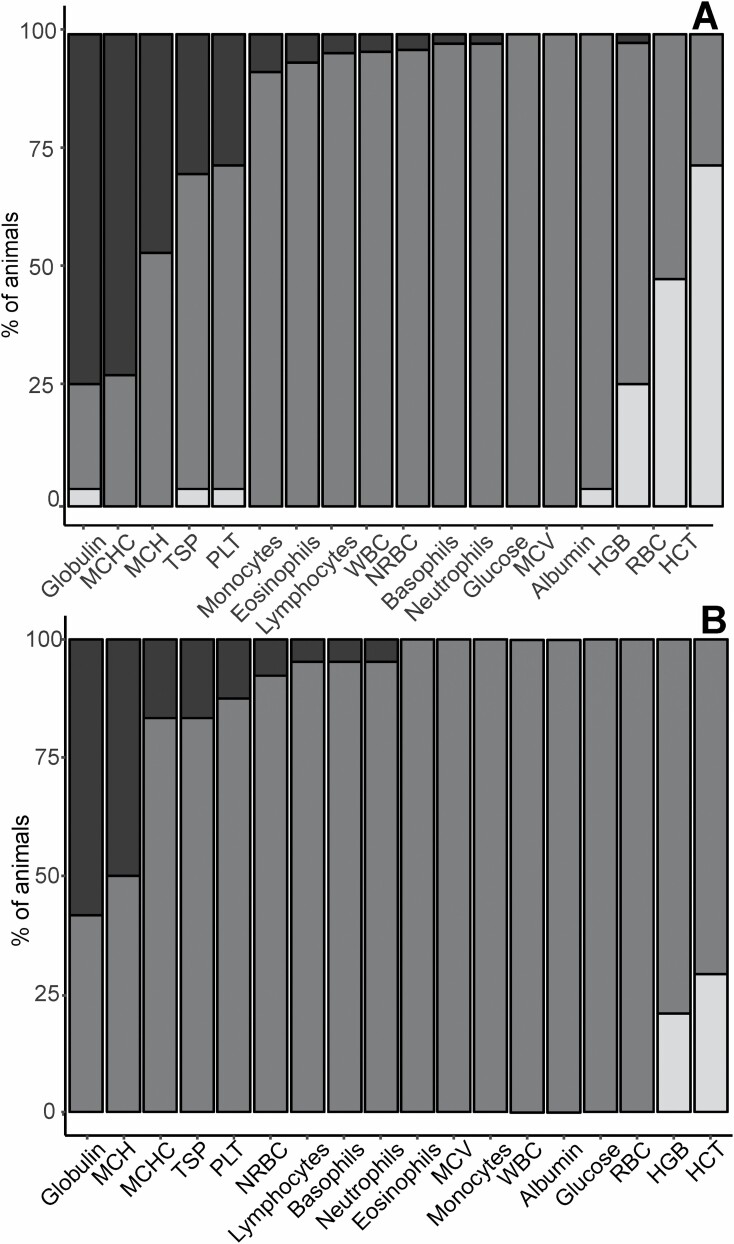
Histograms of the percentage of (A) adult and (B) subadult eastern grey kangaroos (*Macropus giganteus*) whose blood analytes reside within, above, or below the upper and lower limits of the population reference interval (RI) for each blood analyte (Brandimarti et al. 2020). The percentage has been determined from the sample group ((A) *n* ≤ 54 and (B) *n* ≤ 24) at Look at Me Now Headland (LAMN), New South Wales, Australia from 2017 to 2019. Dark gray indicates the percentage of animals sampled within the RI, black and white represent the percentage of animals above or below the reference limits, respectively.

All values were within the species-specific RI for glucose concentration and MCV. As such, these analytes will not be discussed further. There was a greater overall percentage of adults compared to subadults with blood analytes outside the RI limits (Figs. 2A and 2B). More than 90% of adults were within the RI for absolute neutrophil, basophil, lymphocyte, eosinophil, monocyte count, NRBC count, WBC count, and albumin concentration. However, many adults were below the RI for HGB concentration (26%), RBC count (48%), and HCT (72%); and above for PLT count (28%), TSP concentration (30%), MCH (46%), MCHC (72%), and globulin concentration (74%). More than 90% of subadults were within the RI for NRBC count, absolute neutrophil, lymphocyte count, and basophil count. However, many subadults were below the RI for HGB concentration (21%) and HCT (29%); and above the RI for PLT count (13%), MCHC (17%), TSP concentration (17%), MCH (50%), and globulin concentration (58%). Maturity-specific subgroup median values (95% *CI*s) are presented alongside RI upper and lower limits for the LAMN population (Table 2).

**Table 2.** Median (95% confidence intervals, *CI*s) subadult and adult eastern grey kangaroo (*Macropus giganteus*) blood results from the sample group at Look at Me Now Headland (LAMN), New South Wales, Australia from 2017 to 2019. Upper and lower population reference interval (RI) limits are provided for comparison (Brandimarti et al. 2020).

Parameter	Units	Maturity	*n*	Median (95% *CI*s)	Reference interval (*n*)
RBC^a^ count	×10^12^/l	Adult	54	1.5 (1.4, 1.7)	1.49–4.51 (178)
		Subadult	24	1.8 (1.5, 2.2)	0.46–6.27 (54)
WBC^b^ count	×10^9^/l	Adult	54	6.9 (6.4, 7.5)	1.78–11.88 (173)
		Subadult	24	6.5 (5.6, 7.6)	2.28–15.09 (53)
Glucose	mmol/l	Adult	52	3 (2.8, 3.2)	1.09–5.47 (98)
		Subadult	22	3.6 (3.3, 4)	1.31–6.77 (35)
Neutrophil count	×10^9^/l	Adult	50	2.8 (2.5, 3.2)	0.62–5.24 (155)
		Subadult	21	1.8 (1.5, 2.4)	0–4.1 (42)
Lymphocyte count	×10^9^/l	Adult	50	3.3 (3, 3.7)	0.74–7.59 (161)
		Subadult	21	4.1 (3.4, 5.1)	0.09–8.56 (40)
Eosinophil count	×10^9^/l	Adult	50	0.7 (0.5, 0.8)	0.04–1.39 (155)
		Subadult	21	0.4 (0.2, 0.5)	0–1.1 (39)
Monocyte count	×10^9^/l	Adult	50	0.2 (0.1, 0.3)	0–0.43 (155)
		Subadult	21	0.2 (0.1, 0.3)	0–0.66 (40)
Basophil count	×10^9^/l	Adult	50	— ^l^	0–0.08 (155)
		Subadult	21	— ^l^	0–0.09 (40)
HGB^c^	g/l	Adult	54	114 (108, 120)	98.8–164 (175)
		Subadult	24	105 (97, 112)	88.28–162.52 (51)
HCT^d^	l/l	Adult	54	0.1 (0.1, 0.1)	0.15–0.41 (179)
		Subadult	24	0.2 (0.1, 0.2)	0.12–0.47 (54)
MCV^e^	fl	Adult	54	84 (82.2, 85)	61.82–108.35 (178)
		Subadult	24	81.3 (79.3, 83.6)	60.95–112.17 (54)
MCH^f^	pg	Adult	54	78 (71, 86)	29.94–75.62 (179)
		Subadult	24	62.2 (52.1, 74)	15.06–60.72 (53)
MCHC^g^	g/l	Adult	54	944 (858, 1038)	352.13–793.64 (180)
		Subadult	24	754 (639, 907)	315.47–1132.14 (54)
PLT^h^	×10^9^/l	Adult	54	196 (165, 224)	74.38–259.55 (168)
		Subadult	24	141 (105, 184)	11.43–278.87 (51)
NRBC^i^	×10^9^/l	Adult	30	— ^l^	0–0.16 (45)
		Subadult	13	— ^l^	0–0.06 (26)
Albumin	g/l	Adult	27	28.8 (27.2, 30.2)	16.28–42.27 (68)
		Subadult	12	28.4 (26, 32)	22.32–41.53 (32)
TSP^j^	g/l	Adult	27	76.8 (73, 80.3)	59.14–80.99 (31)
		Subadult	12	69.8 (68.1, 76.8)	48.75–77.85 (26)
Globulin	g/l	Adult	27	47.3 (43.9, 50.9)	24.35–41.76 (30)
		Subadult	12	41.8 (35.4, 48)	19.63–40.24 (26)
RET^k^	×10^9^/l	Both	31	(2 counted in *n* = 31)	1.0–1.0

^a^Red blood cell count, ^b^white blood cell count, ^c^hemoglobin, ^d^hematocrit, ^e^mean corpuscular volume, ^f^mean corpuscular hemoglobin, ^g^mean corpuscular hemoglobin concentration, ^h^platelets, ^i^nucleated red blood cell count, ^j^total serum protein, ^k^absolute reticulocyte count, ^l^values too low.

Rainfall had a significant effect on absolute lymphocyte (χ ^2^_1_ = 6.59, *P* < 0.05), eosinophil (χ ^2^_1_ = 5.32, *P* < 0.05), and monocyte counts (χ ^2^_2_ = 7.78, *P* < 0.05). There was a general trend for decreasing absolute lymphocyte and eosinophil count with increasing rainfall; however, the opposite was true for absolute monocyte count. There were no significant effects on any other blood parameters.

#### Blood smears and N:L ratio

**Fig. 3.**
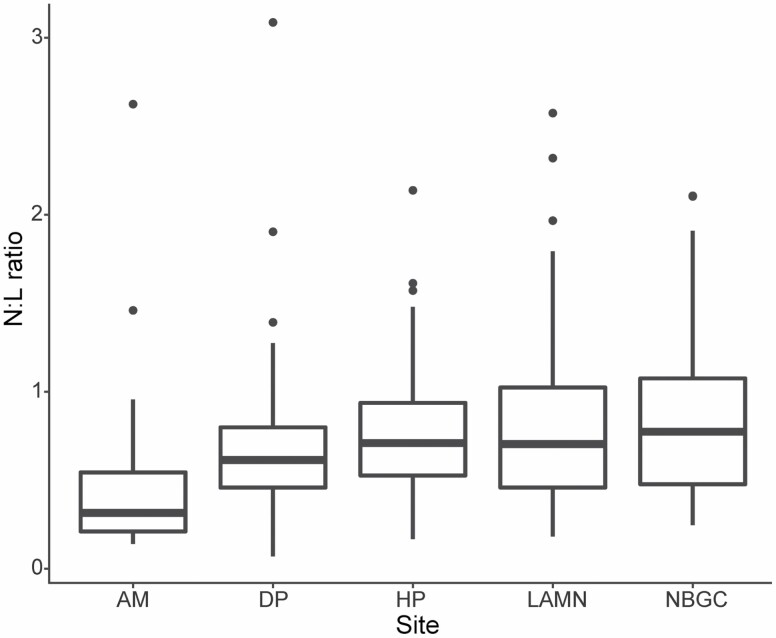
Box and whisker plot of the relative proportion of absolute neutrophils to absolute lymphocytes (N:L ratio) from eastern grey kangaroos (*Macropus giganteus*) sampled from Look at Me Now Headland (LAMN, *n* = 71) from 2017 to 2019, Nelson Bay Golf Course from 2015 to 2019 (NBGC, *n* = 81), Darlington Park (DP, *n* = 47) from 2017 to 2019, Heritage Park (HP, *n* = 62) in 2017, and Ainslie Majura Kangaroo Management Unit (AM, *n* = 25) in 2018. Box plots display the median, with the lower and upper limits of the box corresponding to the 25th and 75th percentiles. The upper whisker extends to the largest value no further than 1.5 times the interquartile range. The lower whisker extends to the smallest value at most 1.5 times the interquartile range. Data beyond the end of the whiskers are outliers and are plotted individually.

Microcytosis (reduced RBC size), hypochromasia (pale cytoplasmic color), and target cells (RBC surface disproportionate to volume) were noted in several blood smears. Two reticulocytes were found from examination of *n* = 31 reticulocyte smears from the sample group at LAMN (Table 2). N:L ratio differed significantly among sites (χ ^2^_4_ = 33.43, *P* < 0.001) and maturity (χ ^2^_1_ = 46.75, *P* < 0.001). NBGC had the greatest mean (± *SD*) N:L ratio (0.83 ± 0.42) followed by LAMN (0.82 ± 0.5), HP (0.77 ± 0.38), DP (0.71 ± 0.49), and AM (0.5 ± 0.54; Fig. 3). These differences were significant for AM and each of LAMN, HP, and NBGC (*P* < 0.05 for all three) as determined by estimated marginal means. Across all sites, adults had significantly greater N:L ratios compared to subadults (0.84 ± 0.46, *n* = 225 and 0.5 ± 0.37, *n* = 61, respectively).

### Trace Element and Heavy Metals

Median (95% *CI*s) trace element and heavy metal concentrations at LAMN were compared to two other kangaroo populations (Table 3). Based on qualitative comparison of the data (due to the limited sample size at NBGC and WGA), there was a higher mean concentration of nickel, tin, mercury, cadmium, and bismuth, at LAMN compared to NBGC and WGA. Kangaroos from LAMN had a lower concentration of iron, selenium, zinc, and to a lesser extent magnesium, compared to NBGC and WGA. WGA had the highest concentration of manganese, selenium, and lead. Arsenic concentration was highly variable among all three sites; however, it was highest at WGA and below the detection limit at NBGC.

**Table 3.** Median (95% confidence intervals, *CI*s) eastern grey kangaroo (*Macropus giganteus*) blood trace and heavy metal concentration (µg/l) from three different sites in 2019; Look at Me Now Headland (LAMN), Nelson Bay Golf Course (NBGC), and Woolgoolga (WGA), New South Wales, Australia.

Analyte (µg/l)	LAMN (*n* = 33)	NBGC (*n* = 5)	WGA (*n* = 2)
Mg^a^	36314 (35519, 37189)	37806 (33328, 42283)	37920 (37608, 38230)
Mn^b^	25.3 (21.7, 30.2)	23.4 (11.2, 45.1)	40.8 (37.1, 44.6)
Co^c^	0.6 (0.5, 0.7)	0.5 (0.3, 0.7)	0.6 (0.4, 0.7)
Ni^d^	2.1 (1.8, 2.4)	1.3 (0.9, 2)	1.7 (1.3, 2.1)
Zn^e^	2275 (2216, 2332)	2553 (2239, 2866)	2462 (2401, 2523)
As^f^	120 (107, 131)	BDL^s^	541 (489, 594)
Al^g^	118 (97.4, 140.9)	132 (104, 168)	115 (102, 129)
Cr^h^	9.3 (9.1, 9.6)	9 (8.3, 12)	9.3 (9, 10)
Fe^i^	370991 (352749, 388513)	494037 (463713, 535697)	445786 (402065, 489507)
Cu^j^	344 (320, 370)	359 (346, 414)	362 (352, 372)
Se^k^	74.4 (54.2, 100)	91.6 (68.8, 114.4)	373 (352, 395)
Cd^l^	0.2 (0.1, 0.3)	0.1 (0, 0.5)	0.2 (0.1, 0.2)
Sn^m^	0.7 (0.6, 0.8)	0.4 (0.3, 0.6)	0.6 (0.5, 0.7)
Sb^n^	14.3 (12.3, 16.7)	12.5 (10.2, 15.3)	12.7 (12, 13.5)
Hg^o^	8.6 (5.4, 14)	1.4 (1.1, 1.7)	2 (1.6, 2.5)
Tl^p^	0.1 (0.1, 0.2)	0.1 (0, 0.1)	0.1 (0.1, 0.1)
Pb^q^	16.6 (14.3, 18.8)	7.4 (5.1, 8.6)	24 (22.2, 25.8)
Bi^r^	0.2 (0.1, 0.2)	0.1 (0, 0.1)	0.1 (0.1, 0.2)

^a^Magnesium, ^b^manganese, ^c^cobalt, ^d^nickel, ^e^zinc, ^f^arsenic, ^g^aluminium, ^h^chromium, ^i^iron, ^j^copper, ^k^selenium, ^l^cadmium, ^m^tin, ^n^antimony, ^o^mercury, ^p^thallium, ^q^lead, ^r^bismuth, ^s^below detection limit.

### Parasitological Results

**Fig. 4.**
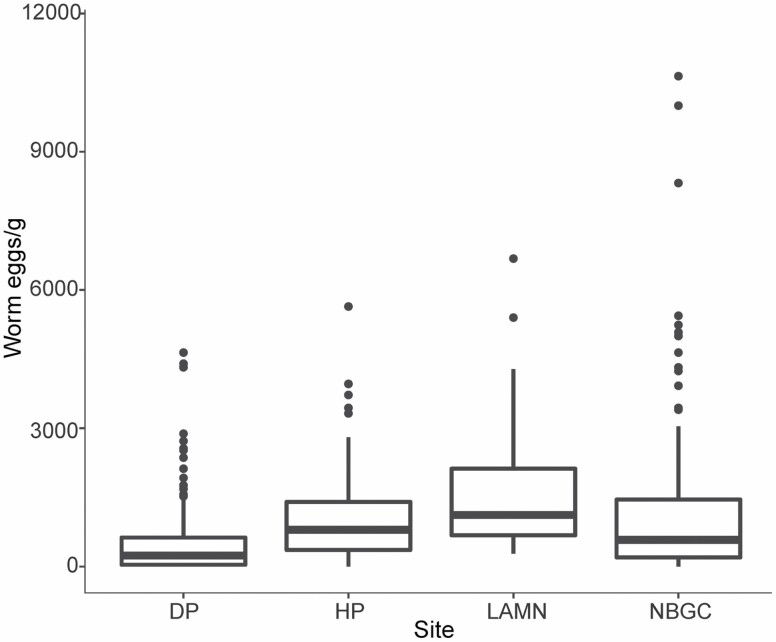
Box and whisker plot of worm eggs per gram of feces (EPG) from eastern grey kangaroos (*Macropus giganteus*) sampled from Look at Me Now Headland (LAMN, *n* = 45) from 2017 to 2019, Nelson Bay Golf Course (NBGC, *n* = 172) from 2015 to 2019, Darlington Park (DP, *n* = 162) from 2017 to 2019, and Heritage Park (HP, *n* = 39) in 2017. Box plots display the median, with the lower and upper limits of the box corresponding to the 25th and 75th percentiles. The upper whisker extends to the largest value no further than 1.5 times the interquartile range. The lower whisker extends to the smallest value at most 1.5 times the interquartile range. Data beyond the end of the whiskers are outliers and are plotted individually.

**Fig. 5.**
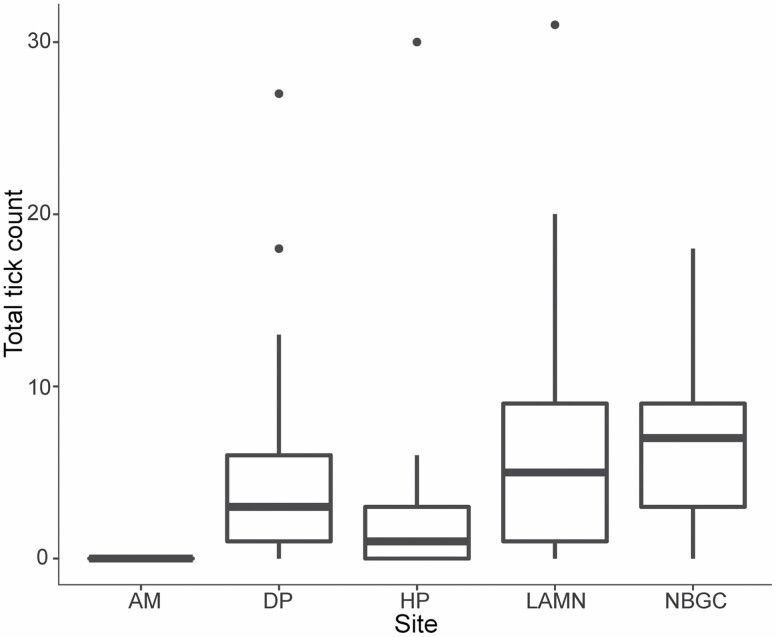
Box and whisker plot of total tick counts from eastern grey kangaroos (*Macropus giganteus*) sampled from Look at Me Now Headland (LAMN, *n* = 79) from 2017 to 2019, Nelson Bay Golf Course (NBGC, *n* = 54) from 2015 to 2019, Darlington Park (DP, *n* = 62) from 2017 to 2019, Heritage Park (HP, *n* = 64) in 2017, and Ainslie Majura Kangaroo Management Unit (AM, *n* = 28) in 2018. Box plots display the median, with the lower and upper limits of the box corresponding to the 25th and 75th percentiles. The upper whisker extends to the largest value no further than 1.5 times the interquartile range. The lower whisker extends to the smallest value at most 1.5 times the interquartile range. Data beyond the end of the whiskers are outliers and are plotted individually.

EPG differed significantly among sites (χ ^2^_3_ = 22.97, *P* < 0.001). Kangaroos at LAMN had the highest EPG values (1660 ± 1444), followed by HP (1279 ± 1343), and NBGC (1173 ± 1644), all of which were significantly greater than DP (549 ± 834) as determined by estimated marginal means (Fig. 4). Total tick counts differed significantly among sites (χ ^2^_4_ = 24.34, *P* < 0.001) and sex (χ ^2^_1_ = 10.66, *P* < 0.01). Across all sites, total tick counts were higher in males (5.03 ± 5.6, *n* = 115) compared to females (3.96 ± 5.26, *n* = 172). Kangaroos at NBGC had the greatest mean total tick count (6.48 ± 4.48), followed by LAMN (6.42 ± 6.63), DP (4.39 ± 4.95), HP (2.05 ± 3.91), and AM (0 ± 0; Fig. 5). Estimated marginal means showed that this difference was significant for LAMN and HP (*P* < 0.01) and DP and HP (*P* < 0.05). Mite presence–absence differed significantly among sites (χ ^2^_2_ = 6.97, *P* < 0.05), seasons (χ ^2^_3_ = 9.35, *P* < 0.05), and between sexes (χ ^2^_1_ = 7.62, *P* < 0.01). Mites were seen more commonly in females (25%) compared to males (6%). Estimated marginal means showed a significant difference in occurrence of mites between two sites: HP and LAMN (*P* < 0.05). Mites were seen more commonly in kangaroos sampled at LAMN (38% of the sample population), followed by DP (14%), and HP (2%). Mite presence–absence also was significantly different among seasons, as determined by post hoc testing; spring accounted for 39% of mite observations, followed by summer (25%), autumn (11%), and winter (7%).

### Body Condition

A subjective body condition score was assigned to *n* = 30 animals from LAMN. Eleven animals were in “good” condition (*n* = 7 adults and *n* = 4 subadults), 12 animals were in “thin” condition (*n* = 8 adults and *n* = 4 subadults), and seven animals were in “poor” condition (*n* = 4 adults and *n* = 3 subadults). No animals were assessed as being in “excellent” condition.

## Discussion

This study highlights several health concerns in the LAMN kangaroo population that were not apparent in other populations existing at lower densities. Principal among these concerns is the significant number of animals with blood analytes outside of the species RI and high parasite loads. Another concern is the number of animals in “thin” and “poor” body condition. We hypothesize that high animal density at LAMN is associated with nutritional stress and higher parasite prevalence, which in turn has a direct influence on hematological parameters. Densities of more than two kangaroos per ha are considered high (Banks et al. 2000) and all of our comparative populations in which health concerns have not been identified are below this figure. The population density at LAMN (5.4 individuals per ha) is more than double that of many densities previously recorded for this species (Southwell 1984; Banks et al. 2000; Ramp and Coulson 2002; Brandimarti et al. 2020). Management of further population growth will be critical for the long-term improvement of health and welfare in this population. However, veterinary intervention on an “as needed” basis is recommended in circumstances of animals showing obvious signs of disease, poor condition, and compromised welfare.

A large number of adults and a lesser number of subadults sampled at LAMN were anemic based on RI comparisons. Decreased HCT, RBC counts, and HGB concentration were noted, consistent with clinical observations of mucous membrane pallor in sampled individuals. Anemia can be characterized either as regenerative or nonregenerative based on the number and proportion of circulating immature RBCs (reticulocytes and NRBC) and manifests clinically as lethargy, reduced exercise tolerance, and impaired weight gain and growth (Canale and Lanzkowsky 1970; Thrall et al. 2012).

Anecdotal observations at LAMN indicated that many single kangaroos appeared less active (lethargic) and had self-isolated from the larger group of kangaroos, which is unusual for a gregarious animal. Based on the absence of reticulocytes (except for two in total across all animals sampled), the anemia observed is classified as nonregenerative, such that the HCT would not return to the normal RI if the disease impact is ongoing (Neiger et al. 2002; Thrall et al. 2012). Blood smears revealed evidence for nonregenerative anemia including hypochromasia, target cells, and microcytosis. Microcytosis, however, was not reflected in the automated hematological results. The small size of microcytic RBCs can result in undercounting by automated cell counters, resulting in falsely elevated MCV calculations (Weiss 2010), which could explain this disparity. Nonregenerative anemia is due to defective or reduced production of RBCs in the bone marrow (Thrall et al. 2012) and can be caused by primary bone marrow disease, reduced availability of iron, or secondary impacts on the bone marrow due to anemia of chronic disease (Nemeth 2008). There are a number of chronic conditions that cause secondary anemia, including: inflammation, renal disease, neoplasia, and endocrine abnormalities; anemia due to chronic parasitism (generally due to hemorrhage) and nutritional anemias are also recognized (Weiss 2010). The effect of nutritional status on RBC counts in kangaroos was partly addressed by Portas and Snape (2018); however, investigation of the underlying cause of the anemia in this population is critical. Thrombocytosis (platelet number above the RI) also was seen in 28% of adults and 13% of subadults. Thrombocytosis is a common finding in acute blood loss and/or iron deficiency anemia and has been observed in many animal species (Weiss 2010; Helmick and Milne 2012; Lee McMichael et al. 2015).

Age-related differences in blood parameters also were evident. Subadult kangaroos from LAMN had lower RBC indices (MCV, MCH, and MCHC) compared to adults, although interestingly, most of these indices either were within or above the RI. The greater percentage of adults diagnosed with anemia could reflect the chronic nutritional deficiencies at this site. Given the greater body size of adults and the additional energy expenditure of lactating females, the nutritional requirements of adults are greater than those of subadults (Munn and Dawson 2001). Subadult kangaroos have an energy requirement approaching 70% of an adult female; therefore, it is likely that the health of subadults will decline at weaning and there may be long-term impacts on growth with increased susceptibility to disease in adulthood (Munn and Dawson 2001; Huynh et al. 2015).

Rainfall has been shown to play a large role in determining erythrocyte values (Brandimarti et al. 2020). For example, the relationship between RBC count and rainfall is parabolic, with a decrease in RBC counts at 100 mm of rain (Brandimarti et al. 2020). In this study there was no significant effect of rainfall on any erythrocyte values, only on differential WBCs. Nutritional stress caused by competition for food alongside prolonged drought may have masked the short-term effect of rainfall on erythrocytes, which was measured over less than 6 months.

N:L ratios have been used recently in wildlife populations as an indirect measure of stress (Davis et al. 2008). In response to stress, the number of neutrophils increases, while the number of lymphocytes decreases (Davis et al. 2008). Significantly higher N:L ratios found in kangaroos at the peri-urban sites LAMN, NBGC, and HP, compared to AM (a nature reserve) could indicate populations under stress. Previous research in Queensland established that kangaroos living within the peri-urban space had higher levels of stress hormone metabolites than their rural counterparts (Brunton et al. 2020). However, the opposite was true for populations in the Australian Capital Territory (Brunton et al. 2020). In our study, it was not possible to delineate the impacts of anthropogenic stress from other causes such as nutritional stress, high density, and parasitism.

Many kangaroos at LAMN had TSP concentrations above the RI. Increased protein often results from hemoconcentration (Senay and Christensen 1965), and prolonged skin “tenting” was present in several individuals, indicating dehydration. Albumin concentrations, however, were within the normal RI, suggesting that a component of the elevated TSP concentration can be attributed to a true increase in globulins. In most animals, globulin concentrations were above the RI. Globulin concentrations can increase in response to inflammation and infection (Serrano et al. 2018) as well as prolonged immunostimulation due to underlying disease. This increase could be attributed to chronic parasitism in this population, which is increased in adults who spend more time grazing to meet their energy requirements. Differentiation of the globulin fractions by serum protein electrophoresis would aid interpretation of globulin results.

Kangaroos (among others in the Macropodoidea) have been known to host the most taxonomically diverse array of parasites of any known mammalian group (Beveridge and Chilton 2001), many of these parasite taxa have been documented previously (Beveridge and Arundel 1979; Arundel et al. 1990; Vogelnest and Portas 2008). The impact of parasitism can range from no effect, to reduced growth and reproduction, clinical disease, and mortality; depending on the species and parasite load (Gulland 1995). Nematode species such as *Rugopharynx rosemariae* have been known to cause severe hypertrophic gastritis (inflammation of stomach mucosa) in kangaroos, while large burdens of *Globocephaloides trifidospicularis* may cause hemorrhage, anemia, and mortality in juvenile kangaroos (Arundel et al. 1990; Fazenda 2009).

Kangaroos at LAMN had a higher median EPG (range 280–6680) compared to any other site in this study; however, the range overlaps with NBGC and HP. The high EPG at LAMN also exceeds those reported in studies of other kangaroo populations (Cripps et al. 2013, 2014, 2015). The high population density at LAMN is a likely driver of parasitism because the high contact rate among kangaroos promotes parasite transmission (Gortázar et al. 2006). High population density has been suggested to increase parasitism in other marsupial species, such as the Tasmanian devil (*Sarcophilus harrisii*—Gregory et al. 1975; Arneberg et al. 1998). The mechanistic model for this pathway is that as host density increases, each parasite larva or egg has an increased chance of contacting a host (May and Anderson 1978). Despite being a common and nonlethal practice for estimating nematode burdens, EPGs from LAMN should be interpreted with caution as they may not be representative of an animal’s total worm burden, rather driven by a single species of nematode present (Cripps et al. 2015).

**Fig. 6.**
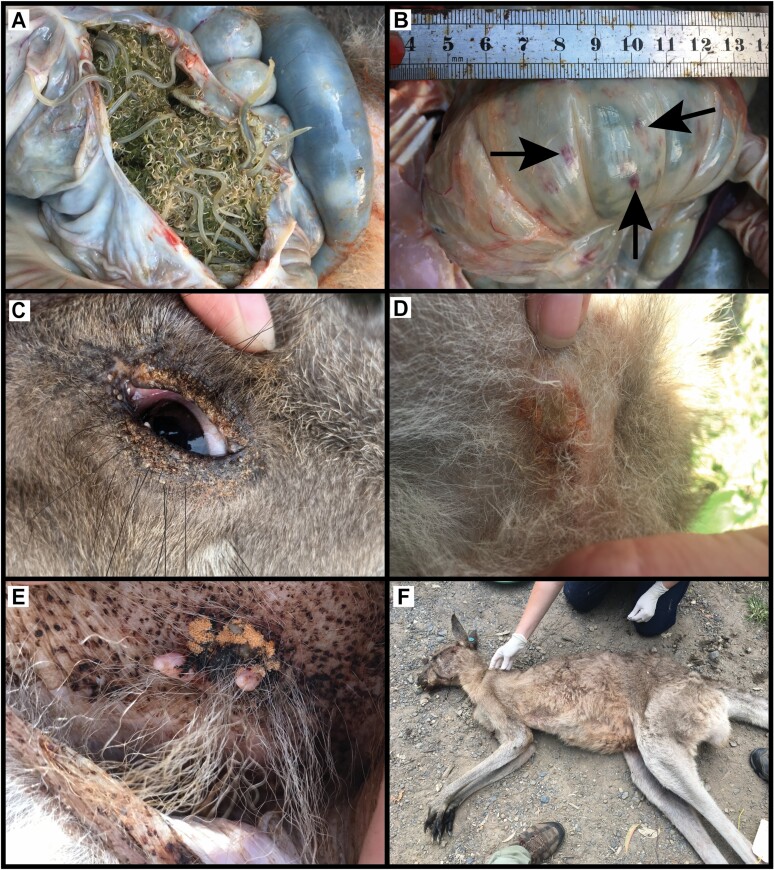
(A) Large gastrointestinal worm burden in the stomach. (B) Multifocal hemorrhages (indicated by arrows) of the gastric wall. (C) Tick and mites around the perimeter of the eye. (D) Orange patches associated with pruritis and inflammatory lesions of the axilla. (E) Mites and inflammation at the base of the teat in a female kangaroo. (F) Emaciated male kangaroo dispatched by police due to reports by the public of an animal with severe emaciation and lethargy. All eastern grey kangaroos (*Macropus giganteus*) photographed were from Look at Me Now Headland (LAMN) during the study period (2017 to 2019).

Opportunistic necropsy of kangaroos from LAMN revealed large gastric nematode burdens (Fig. 6A), accompanied by multifocal hemorrhages of the gastric wall (Fig. 6B). Scouring also was noted in some individuals. A limitation of our investigation was the lack of assessment of species; hence, assessment of proportion of parasite species present. For this reason, the impact of gastric parasitism on the health status and blood parameters of this population is not able to be quantified. However, given the high occurrence of nonregenerative anemia in the LAMN population, as well as increased eosinophil counts in some adult kangaroos, it is possible that pathogenic nematode species could be contributing to the poor health of kangaroos at LAMN. Identifying the parasites present is a recommended next step for future research.

External parasitism also was evident at LAMN, with significantly higher total tick counts compared to most other sites (Fig. 6C), with a significant tick bias in males compared to females. This bias has been firmly established in many studies and is thought to occur because of the immunomodulatory effects of testosterone in males (Folstad and Karter 1992; Zuk et al. 1995; Tschirren et al. 2003; Pollock et al. 2012).

Kangaroos from LAMN had a higher incidence of mites, with 38% of the sample population having mite infestations. Larvae of the family Trombiculidae have been described in the agile wallaby (*Notamacropus agilis*) and bridled nailtail wallaby (*Onychogalea fraenataies*), and have been found to cause inflammatory lesions (Turni and Smales 2001; McLelland and Youl 2005; Old et al. 2009). Clinical examination of affected animals showed orange patches associated with pruritis and inflammatory lesions of the axillae (Fig. 6D), inguinal region, perimeter of the eyes, and in the inner ear-folds (Mullen and O’Connor 2019). There was a significantly higher incidence of mites in female kangaroos at LAMN, and often mites were found inside the pouch, attached to and around the base of the teats, which appeared reddened and swollen (Fig. 6E). This sex difference could be attributed to the warm, humid, and protected location of the pouch, which benefits mites and allows close contact with the skin. There also was a higher incidence of mites in summer, when the climate at LAMN is hot and humid. Similar environmental conditions have been promoted as a likely cause of increased mite numbers in the bridled nailtail wallaby (Old et al. 2009). Kangaroos at LAMN were found to have the greatest incidence of mites compared to other local sites (DP and HP).

Body condition can indicate nutritional status and is intrinsically linked to parasite burden (Ndlovu et al. 2007; Sánchez et al. 2018). Most kangaroos sampled from LAMN were in “thin” (40%) or “poor” (23%) body condition and had a dull coat (Fig. 6F) as well as pale oral and urogenital mucous membranes, a clinical finding consistent with anemia. This study was unable to compare body condition with lower density sites; however, most sampled animals scored in the lowest two categories (Johnston et al. 1997). A high intensity of grazing pressure has been reported at LAMN (Hunter and Hunter 2019). Given the high density of kangaroos and their strong site fidelity, it is hypothesized that kangaroos are experiencing nutritional stress and, as a result, may not be able to mount an adequate immune response to their parasite burdens. A lack of micronutrients, particularly zinc, selenium, and iron, all of which were lower in animals from LAMN compared to other sites studied, can lead to clinically significant immune deficiency (Cunningham-Rundles et al. 2005). An adequate immune response in kangaroos also could be inhibited by increased concentration of mercury and cadmium, both of which are nonessential elements and can cause immunotoxicity (Moszczyński 1997; El-Boshy et al. 2015).

**Fig. 7.**
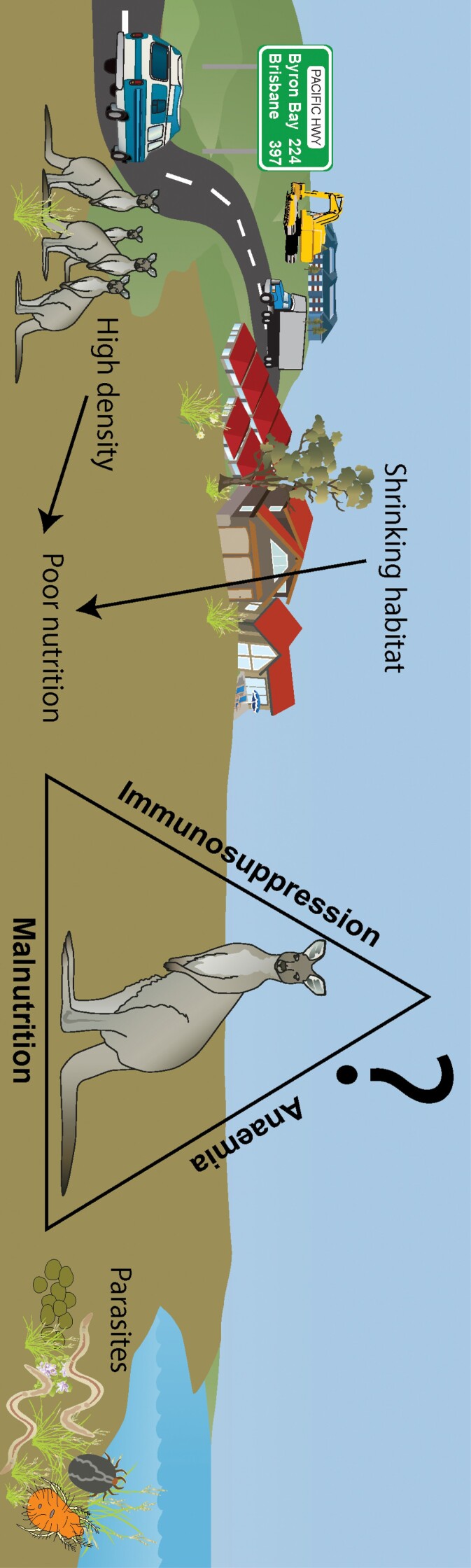
Hypothesized factors contributing to poor health and welfare of eastern grey kangaroos (*Macropus giganteus*) at Look at Me Now Headland (LAMN). Symbols courtesy of the Integration and Application Network, University of Maryland Center for Environmental Science (https://ian.umces.edu/symbols/).

Our study determined that the LAMN kangaroo population had one of the highest population densities recorded for this species, with evidence of chronic nutritional stress, parasitemia, and overall poor welfare (Fig. 7). These issues likely are a consequence of human-induced environmental modification and for this reason, management of this population is recommended. Short- and long-term management strategies, concurrent with ongoing health and disease monitoring, should be employed to ensure improved welfare outcomes for individuals. Short-term strategies could include lethal control (culling) which will reduce animal density but could be a controversial option and lacking in local support. A targeted approach, whereby a veterinarian selects individuals with compromised welfare for humane euthanasia, based on signs of ill-thrift, and poor body condition and associated symptoms (lethargy, reluctance to move), would achieve both improved animal welfare outcomes and reduced population density. Once the population size is reduced, reproductive management could be used as a longer-term solution to manage population growth. Reproductive management also could improve the health status of female kangaroos, reducing the metabolic demands of a pouch young and lactation (Gélin et al. 2015). In the long term, local and regional planning must prioritize the maintenance of habitat connectivity for kangaroo populations to prevent overabundance and enhance animal health and welfare. This study has demonstrated how local overabundance can result in concerning animal welfare outcomes. Future coastal developments must incorporate landscape ecology measures (Collinge 1996) such as wildlife movement corridors that may alleviate some pressures on urban kangaroos; however, swift management interventions at LAMN are required to improve the health and welfare outcomes for this population.

## Supplementary Data

Supplementary data are available at *Journal of Mammalogy* online.

Supplementary Data SD1.—Foot based eastern grey kangaroo (*Macropus giganteus*) count transect of Damerells and Look at Me Now Headland, New South Wales, Australia.

gyab022_suppl_Supplementary_Data_SD1
