# Supplementary material for: Kangaroos at maximum capacity: health assessment of free-ranging eastern grey kangaroos on a coastal headland
Source: J Mammal. 2021 Mar 30;102(3):837–51. doi: 10.1093/jmammal/gyab022 (PMC8355480; doi:10.1093/jmammal/gyab022)
Supplement: gyab022_suppl_Supplementary_Data_SD1 [file gyab022_suppl_supplementary_data_sd1.docx]

**SUPPLEMENTARY DATA**


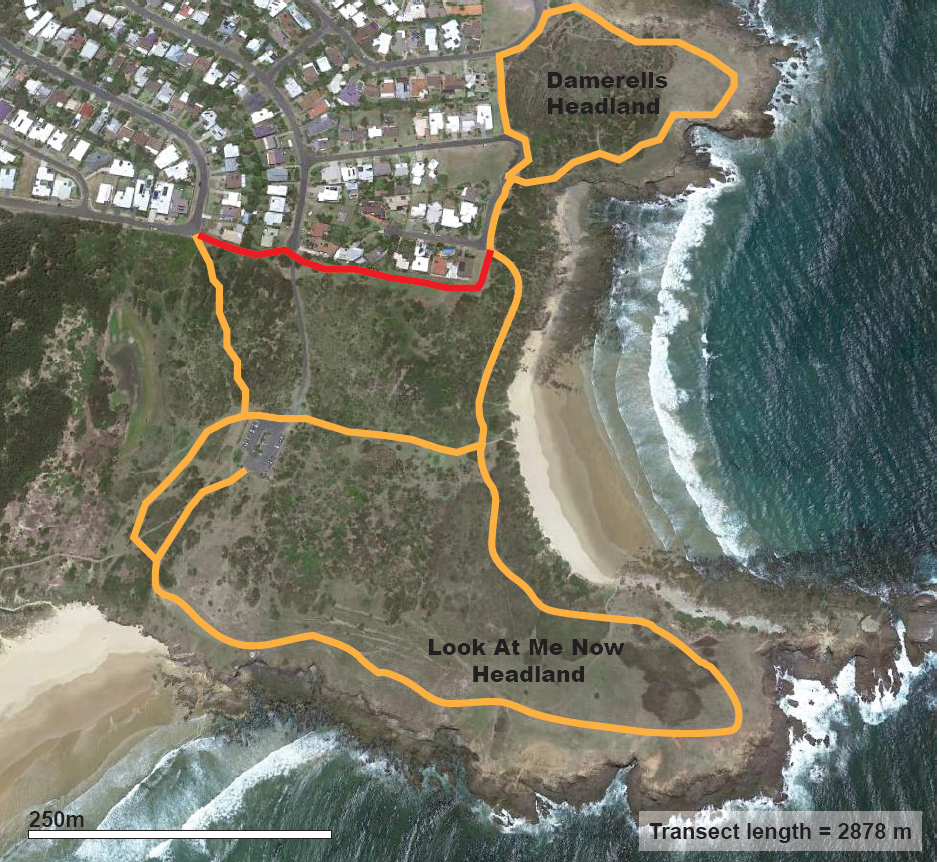


Supplementary Data SD1. - Foot based eastern grey kangaroo (*Macropus giganteus*) count transect of Damerells and Look at Me Now Headland, New South Wales (NSW), Australia. The existing count transect (Henderson et al. 2018) is yellow; the red line denotes the additional transect line utilised in this study.
